# Data on thermal infrared imaging in laboratory non-human primates: Pleasant touch determines an increase in nasal skin temperature without affecting that of the eye lachrymal sites

**DOI:** 10.1016/j.dib.2016.09.029

**Published:** 2016-09-23

**Authors:** Laura Clara Grandi, Eugenio Heinzl

**Affiliations:** a Università di Parma, Dipartimento di Neuroscienze, 43125 Parma, Italy; b Università di Milano, Dipartimento di Medicina Veterinaria DiMeVet, 20133 Milan, Italy

**Keywords:** Infrared thermography, Non-human primates, Pleasant touch, Heart rate, Heart rate variability

## Abstract

The data described here relate to the article entitled “The effect of pleasant touch on nose skin temperature, heart rate and heart rate variability: preliminary results in a male laboratory rhesus monkey” (Grandi and Heinzl, 2016) [1]. The cited paper and article here present additional material which represents the first evidence of the effect of pleasant touch in non-human primates in terms of skin temperature change, as recorded by means of infrared thermography.

The sweep is considered a pleasant touch for monkeys. Here we forward preliminary evidence concerning the modulation of the eye lachrymal site temperature while a monkey received sweeps to the back from a familiar experimenter, and at different speeds (Grandi and Heinzl, 2016) [1].

**Specifications Table**

**Table:** 

Subject area	Autonomous nervous system physiology
More specific subject area	Non-human primate physiology
Type of data	Figures
How data was acquired	Infrared thermography (IRT)
Data format	Analyzed
Experimental factors	A familiar experimenter swept the back of a male rhesus monkey (*Macaca mulatta*) aged five years and weighing 5.6 kg.
	The sweep was performed on the back and at different speeds, as described in [1]. Thermal recordings were conducted using a portable IRT camera (NEC Avio TVS500; Nippon Avionics Co., Ltd., Tokyo, Japan). Details of the software and camera utilized are described in [1].
Experimental features	A male monkey received pleasant touch from a familiar experimenter on the back, and at different speeds. The analyzed physiological parameter was the temperature at the eye lachrymal sites.
Data source location	Parma, Italy
Data accessibility	The data are contained within this article.

**Value of the data**

• The preliminary data presented here are the first evidence of the effect of pleasant touch on the temperature of the eye lachrymal sites in non-human primates.
• The present data correspond to one of the few related studies employing infrared thermography in non-human primates [2], [3], [4]. Therefore, they could represent a starting point to investigate the autonomic effects of pleasant touch by means of a non-invasive technique such as thermal imaging, in both humans and non-human primates.
• The present data, in addition to that shown in [1], are important in terms of animal laboratory welfare, underscoring the positive physiological effect of sweeping [5]. Moreover, they demonstrate that while the nose skin temperature is affected by the touch, the eye lachrymal sites are not. Therefore, further research is necessary in order to deeply investigate the impact of pleasant touch on different sites within the facial region.

## 1 Data

**Fig. 1:**
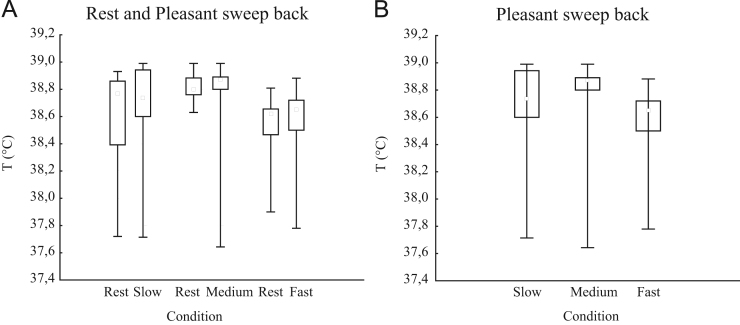
(A) The graph represents the eye lachrymal sites’ temperature (°C) for each pair of rest–slow, rest–medium and rest–fast. (B) The graph represents the eye lachrymal sites’ temperature (°C) to allow comparison among the 3 speeds: slow, medium and fast. In (A) and (B) the small inner gray squares within box of 25th and 75th percentiles represents the median. The whiskers indicate the 10th and 90th percentiles.

The temperature of the eye lachrymal sites (Table 1) while sweeping at slow, medium and fast velocity did not change in comparison to the rest condition, where the monkey did not have any kind of contact with the experimenter (Fig. 1A). The comparison among the three speeds highlights that the eye lachrymal sites failed to present any significant differences between the three stimulations. The temperature during sweep at medium speed showed a small tendency to be higher than during the other two sweeps, but without reaching significance (Fig. 1B).

The correlation analysis (Table 2) revealed that there was no correlation between the room temperature and the monkey׳s eye lachrymal temperature during the rest condition, sweep medium and sweep slow. Instead, there was a correlation between the ambient temperature and sweep fast (medians: sweep fast=38.65 °C; room temperature=25.10 °C. Spearman׳s rank correlation coefficient, *N*=9; *r*_*s*_=−0.84; *p*=.005).

## 2 Experimental design, materials and methods

The recording session consisted of two consecutive phases: (1) the rest condition, during which there was no physical, auditory or visual contact between the monkey and the experimenter; and (2) the sweeping conditions. During the second phase, the experimenter caressed the back of the monkey randomly with one of the three speeds: 1–3 cm/s (slow), 5–10 cm/s (medium) or 16–20 cm/s (fast). In total, we conducted 12 trials for the sweep slow, and 9 trials for each sweep medium and fast. Both the sweeping speeds and the back as the stimulated body part were chosen based on the results of our previous study [5].

We performed four statistical analyses; (1) the Friedman test (*p*<.05) was performed in order to confirm that the three rest conditions do not differ each others; (2) the Wilcoxon test (*p*<.05) was performed in order to compare the three sweep conditions at the three speeds, and the relative pre-stimulation conditions; (3) the Kruskal–Wallis Test (*p*<.05) was performed to compare the sweeping conditions at the three speeds each other; and (4) the Spearman׳s rank correlation analysis, in order to ensure that the temperature of the nasal region was not dependent upon the ambient temperature in the room, for the rest and the three sweeping conditions [2]. For the correlation analysis we compared the ambient temperature with the eye lachrymal sites temperature during the rest condition (sum of the rest prior the sweep at slow, medium and fast speed), the sweep at slow, medium and fast speed. In this analysis we considered the sum of the three rest conditions, since the rest conditions that preceded each sweeping did not differ each others (Friedman Test).

The detailed experimental procedure, as well the data collection and analysis, are described in [1]. Additional to the results of the analysis of the nose skin temperature [1], here we report the results concerning the eye lachrymal sites’ temperature.

## Figures and Tables

**Table 1:** Valid trials (*N*), Mean, Median, Standard deviation for each condition. Slow/medium/fast indicates the monkey׳s eye lachrymal sites temperature during the sweep slow/medium/fast. Rest slow/medium/fast eye indicates the monkey׳s eye lachrymal sites temperature during the rest prior slow/medium/fast sweep.

	***N***	***Mean***	***Median***	***Stand. Dev.***
***Rest slow***	12	38,57	38,77	0,40
***Slow***	12	38,63	38,74	0,43
***Rest medium***	9	38,81	38,80	0,10
***Medium***	9	38,74	38,87	0,42
***Rest fast***	9	38,47	38,62	0,34
***Fast***	9	38,49	38,65	0,41

**Table 2:** Correlation among the room temperature (Room T) during each condition and the monkey׳s eye lachrymal sites temperature in each conditions (Spearman׳s rank correlation). Room T during rest indicates the room temperature during the rest condition (sum of the rest condition of the three sweeping), Room T slow, Room T medium and Room T fast indicate the room temperature during the sweep slow, medium and fast, respectively; Rest T eye indicates the monkey׳s eye lachrymal sites temperature during the rest condition (sum of the rest condition of the three sweeping), Slow/medium/fast eye indicates the monkey׳s eye lachrymal sites temperature during the sweep slow/medium/fast.

	***N***	***Spearman R***	***t*****(*****N*****−2)**	***p*****-*****Level***
***Slow eye T vs Room T slow***	12	a	a	a
***Medium eye T vs Room T medium***	8	−0,38	−1,00	0,36
***Fast eye T vs Room T fast***	9	−0,84	−4,08	***0,005***⁎
***Rest eye T vs Room T rest***	29	0,03	0,16	0,88

⁎ Corr.Sign. *p*<.05 (2-tails).

a The room temperature variable is constant, the analysis is not possible.
