# Supplementary material for: Data on thermal infrared imaging in laboratory non-human primates: Pleasant touch determines an increase in nasal skin temperature without affecting that of the eye lachrymal sites
Source: Data Brief. 2016 Sep 23;9:536–9. doi: 10.1016/j.dib.2016.09.029 (PMC5054237; doi:10.1016/j.dib.2016.09.029)
Supplement: Supplementary file 2 — Supplementary material. [file mmc1.doc]

Conflit of interest: NONE.

As far as we know we don’t have any conflict of interest with any other researcher.

Laura Clara Grandi

Eugenio Heinzl
